# A meta-analysis identifies factors predicting the future development of freezing of gait in Parkinson’s disease

**DOI:** 10.1038/s41531-023-00600-2

**Published:** 2023-12-04

**Authors:** Talia Herman, Yael Barer, Michal Bitan, Shani Sobol, Nir Giladi, Jeffrey M. Hausdorff

**Affiliations:** 1 Center for the Study of Movement, Cognition and Mobility, Neurological Institute, Tel Aviv Sourasky Medical Center, Tel Aviv, Israel; 2 Maccabitech, Maccabi Institute for Research and Innovation, Maccabi Healthcare Services, Tel Aviv, Israel; 3 School of Computer Science, The College of Management, Rishon LeZion, Israel; 4 Sagol School of Neuroscience, Tel Aviv University, Tel Aviv, Israel; 5 Department of Neurology, Faculty of Medicine, Tel Aviv University, Tel Aviv, Israel; 6 Department of Orthopedic Surgery and Rush Alzheimer’s Disease Center, Rush University Medical Center, Chicago, IL USA; 7 Department of Physical Therapy, Faculty of Medicine, Tel Aviv, Israel

**Keywords:** Parkinson's disease, Parkinson's disease

## Abstract

Freezing of gait (FOG) is a debilitating problem that is common among many, but not all, people with Parkinson’s disease (PD). Numerous attempts have been made at treating FOG to reduce its negative impact on fall risk, functional independence, and health-related quality of life. However, optimal treatment remains elusive. Observational studies have recently investigated factors that differ among patients with PD who later develop FOG, compared to those who do not. With prediction and prevention in mind, we conducted a systematic review and meta-analysis of publications through 31.12.2022 to identify risk factors. Studies were included if they used a cohort design, included patients with PD without FOG at baseline, data on possible FOG predictors were measured at baseline, and incident FOG was assessed at follow-up. 1068 original papers were identified, 38 met a-priori criteria, and 35 studies were included in the meta-analysis (*n* = 8973; mean follow-up: 4.1 ± 2.7 years). Factors significantly associated with a risk of incident FOG included: higher age at onset of PD, greater severity of motor symptoms, depression, anxiety, poorer cognitive status, and use of levodopa and COMT inhibitors. Most results were robust in four subgroup analyses. These findings indicate that changes associated with FOG incidence can be detected in a subset of patients with PD, sometimes as long as 12 years *before* FOG manifests, supporting the possibility of predicting FOG incidence. Intriguingly, some of these factors may be modifiable, suggesting that steps can be taken to lower the risk and possibly even prevent the future development of FOG.

## Introduction

Freezing of gait (FOG) is an extremely debilitating problem that is common among people with Parkinson’s disease (PD)^1,2^. FOG is characterized by episodes in which walking cannot start or is interrupted, despite the effort to move forward^2,3^. This severely impairs function, independence, and quality of life^1,4^. FOG is considered the leading contributor to falls in PD, further contributing to its major negative impact^4–6^.

Multiple theories and models have been proposed to explain the causes of FOG^2,7–9^, however, the precise mechanisms that trigger FOG are not yet well understood. Many attempts at ameliorating FOG frequency and severity have also been made. These include anti-parkinsonian medications, cueing, exercise, and invasive and non-invasive brain stimulation^3,10–19^. In addition, interventions designed to ameliorate FOG severity by treating anxiety and depression or improving executive function and other cognitive domains have been investigated^5,11,20–23^. While FOG may partially be resolved in response to these therapies, it generally persists and worsens over time, with minimal or no long-term resolution^2,7,15,18,24^. The sheer number of approaches and unsuccessful studies underscores both the clinical significance of FOG and the absence of an effective therapeutic approach.

To make a robust, clinically meaningful impact, we suggest, therefore, that an “upstream” approach to treating FOG may be needed: prevention. Treating FOG before it occurs has not yet been attempted, likely for several reasons. Prevention requires relatively long, prospective studies. Moreover, it is not yet clear how FOG could be prevented. Still, if modifiable, early signs can be identified, this could, perhaps, lead to the targeted application of FOG prevention among patients who have a greater risk of developing FOG. A growing number of observational studies have prospectively evaluated factors associated with FOG incidence, providing a timely opportunity to review the literature and to start to consider the possibility of preventing or lowering the risk of the development of FOG. Briefly, several factors have been suggested to be associated with FOG incidence. These include, for example, longer disease duration, increased depression or anxiety, higher levels of levodopa daily equivalent dose, reduced sleep quality, worse cognitive function, and the PIGD motor subtype^25–30^. Therefore, in this systematic review and meta-analysis of observational studies, we aimed to summarize and synthesize studies that report on the risk factors and potential early signs of the future development of FOG, among patients with PD who do not yet have FOG.

## Results

### Study selection

**Fig. 1:**
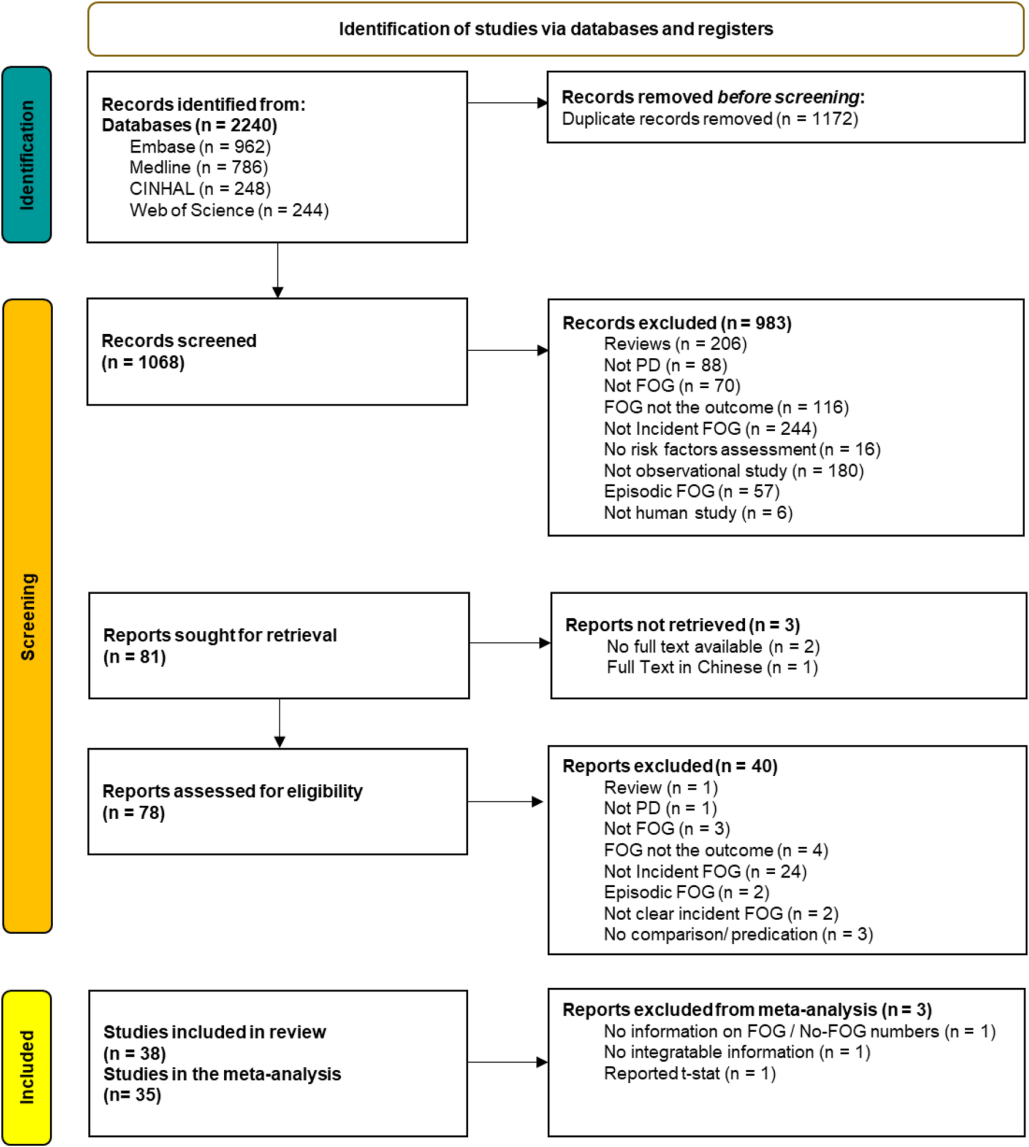
Study selection and PRISMA Flow. Study selection; PRISMA Flow Diagram describing the studies that were initially identified in the search and those that were included in the meta-analysis.

The search yielded 2240 records. After duplicate removal, 1068 distinct records were identified. Of these, 38 were eventually included in the systematic review, and 35 were included in the meta-analysis (Fig. 1). Table 1 summarizes the studies that examined clinical, motor, and non-motor potential predictors and Table 2 summarizes other predictors such as genetic and CSF parameters.

**Table 1:** Clinical, motor, and non-motor predictors of the development of FOG.

Authors, year	Study design	Sample size (*n* total, % who developed FOG)	FOG ascertainment	Follow-up duration (years)	Exposure Predictors	Main findings	Remarks and limitations	Total quality score
Banks et al.^72^	Retrospective cohort	*n* 100, FOG in 50, (50%) matched to no-FOG	self-report	4 years	Non motor features: cognitive, sleep and mood	Baseline processing speed, learning and sleepiness scores were predictive of FOG.	Retrospective analysis, using self-reported FOG to classify patients, investigating FOG development over a relatively short period.	6
Chong et al.^73^	Retrospective cohort	*n* 17 FOG in 11, (65%)	Self-report (old FOG-Q)	2 years	Step initiation duration: Responders Subjects who completed the first step faster with visual cues. Non-responders completed the first step in similar or slower duration.	Decrease in first step duration (with visual cues) is potentially a reliable physical predictor of FOG. The responder group had a 13-fold risk of developing FOG within 2 years. Out of 9 responders, FOG in 8 (89%); out of 8 non-responders, FOG in 3 (38%).	A small and biased sample. Only self-report was used to classify subjects as freezers or non-freezers. Only one physical aspect was tested (i.e., no cognition).	6
Chung Yoo, Lee et al.^28^	Prospective cohort	*n* 257, FOG in 54, (21%)	Self-report OR clinical observation	>2 years	Detailed neuro-psychological test. Global cognitive composite score was calculated based on four factors.	Poorer cognitive performance on visual memory/ visuospatial function- factor 1 & lower global cognitive scores were associated with a higher risk for FOG.	LID, wearing-off, & FOG were not determined quantitatively. Motor deficits were not collected longitudinally. Cognitive domains were not equally represented in the factor analysis.	6
D’Cruz et al.^74^	Prospective cohort	*n* 60, FOG in 12, (20%)	Self-report NFOG_Q	2 years	Multiple motor and non-motor aspects including gait, finger taps, cognition and affect.	Gait asymmetry & inconsistency in scaling & rhythm of repetitive limb movements were worse in future freezers.	Underpowered due to broad predictors. Self-report nature of FOG. Difficult to determine the exact time of conversion.	5
Ehgoetz Martens et al.^25^	Prospective cohort	*n* 75, FOG in 23, (31%)	Self-report N-FOGQ with video	~1.3 year	Motor, LEDD, Cognitive function, Limbic, & Sleep Domains.	TD/non-TD ratio, FOG-Q, TMTB, anxiety, depression, age & disease duration, predicted FOG with 84% accuracy.	Lack of LEDD data. Self-report nature of FOG.	7
Forsaa et al.^42^	Prospective cohort	*n* 169, FOG in 82 (49%).	UPDRS part II #14	12 years	Demographic variables, medical history and medication use, motor and non-motor symptoms.	Risk factors of incident FOG: Higher LEDD, presence of motor fluctuations at baseline and follow-up time. Dyskinesias at baseline were inversely associated with risk for incident FOG. Concomitant features of FOG over time: higher UPDRS motor score, presence of psychosis, higher levodopa LED, and presence of motor fluctuations and dyskinesias. FOG was also positively associated with PIGD severity. For a 100 mg increase in levodopa dose at baseline, the risk of FOG during the 12-year study period increased by 30%.	Follow-up time was a significant predictor of incident FOG, indicating that additional features may be associated with increased FOG risk. Lack of neuropsychological data and quantitative gait assessments. Patients were examined at rather long-time intervals during the first eight years of follow-up. FOG was assessed by history using a single item derived from the UPDRS.	5
Gallea et al.^75^	Prospective cohort	*n* 25, FOG in 17 (68%)	GABS UPDRS	5 years	Parkinsonian signs, cognitive status and oculomotor recordings.	Baseline increased antisaccade latency is a predictive marker of FOG occurrence and onset. The 5-year course of FOG was correlated with worsening antisaccade latencies.	Small number of patients.	5
Garcıa-Ruiz et al.^76^	Prospective cohort	*n* 45, FOG in 31, (69%)	Self-report	10 years	Initial treatment (LD vs no LD), sex, age at onset.	Age at onset, sex, and initial treatment influence the appearance of FOG.	Initial treatment with LD may predispose to motor fluctuations and dyskinesias, but only in the medium term. After the first decade, these differences are no longer relevant.	4
Giladi et al.^77^	Prospective cohort	*n* 743, FOG in 154, (21%)	Self-report (FOG item of UPDRS II ≥ 1)	>1 year	Total UPDRS, disease duration, medication, MMSE, Hamilton depression, gait, balance, and speech.	FOG was strongly associated with gait, balance, and speech problems. Initial motor symptoms on the left side of the body was related to a higher risk of FOG development. Tremor as an initial motor symptom was a protective factor for future FOG. 10 mg Deprenyl reduced the risk of developing FOG by 53%.	Balance as an initial motor symptom was much less prominent as a risk factor for the development of FOG. Later in the course of the disease, falls and abnormal postural reflexes at baseline were significantly associated with FOG. No differentiation between diverse types of speech difficulties.	5
Herman et al.^78^	Prospective cohort	*n* 57, FOG in 26, (46%)	N-FOGQ with video and clinical observation	5 years	Multiple motor and non-motor aspects including gait, balance, MDS-UPDRS, cognition, and affect.	GDS, gait speed & UPDRS-III (on vs. off) were the only significant independent predictors of future FOG.	~80% of subjects with marked depressive symptoms developed FOG during follow up, compared to only 27% in the non-depressed subjects. Anxiety was not assessed. Exact time of conversion is unknown.	6
Jeong et al ^79^	Retrospective cohort	*n* 329, FOG in 52, (16%)	Self-report & clinical observation	≥2 years	Neuropsychiatric inventory included delusions, anxiety hallucinations, depression, apathy, sleep appetite, and eating changes. Grouped into 3 factors: Psychosis, hyperactivity & mood. All subjects had decreased DAT availability in the posterior putamen.	NPI total score was a significant predictor of future FOG within 5 Years (adjusting for confounders). The risk of FOG development during follow up was significantly higher in the NPI (+) group vs. NPI (-).	Assessing the total burden of NPS is crucial for predicting motor outcomes in PD patients. The NPI total score that was used only assesses NPS over the past month (rather than 6 months’ history). Due to retrospective nature, information on longitudinal changes in NPS was lacking. Increase in LEDD may not accurately reflect the progression of PD.	8
Jeong et al.^80^	Retrospective cohort	*n* 571, FOG in 53 males (19%) and 57 females (20%)	History & neurological examination	6 years	Gender	FOG development did not differ according to sex.	Baseline DAT was used as a covariate in all analyses. Occurrence of motor complications was determined based on history and neurological examination without any quantitative analyses. The study has a female predominance which is inconsistent with Western epidemiology. It was difficult to accurately evaluate the effect of estrogen exposure on nigrostriatal dopaminergic degeneration owing to the lack of information on menstruation or menopause in female patients with PD.	7
Kelly et al.^81^	Retrospective cohort	*n* 783, FOG incidence is not reported	MDS-UPDRS item 3.11	4 years	A detailed neuropsychological assessment, including global cognition, executive function, memory, visuospatial function, and language.	Deficits in global cognition (MoCA and Mattis Dementia Rating Scale-2), digit symbol and visuospatial impairments were associated with higher risk for FOG.	The cross-sectional nature does not support causation. Cognitive domains differ across studies and the tests utilized here differ from some previous research. Medications were not included as a covariate in the adjusted models.	8
Kim, Jeon et al.^82^	Retrospective cohort	*n* 1212, FOG in 121, (10%)	Neurological exam and history taking	~2 years	Demographic and clinical information.	An older age at onset (≥60 years) was a predictor for FOG. FOG showed an incidence of 1.5% at the 5-year, 10.3% at 10-year and 23.7% at 15-year PD.	There was no duration of levodopa use as a time factor, but the disease duration which might indicate lower prevalence. The age at PD onset is younger than the usual PD population. Follow up interval was not the same for all participants. Possible recall bias since onset of motor complication was assumed as the time it was observed.	8
Lee et al.^83^	Retrospective cohort	*n* 108, FOG in 31, (28.7%)	Self-report	~3 years	Korean version of “Sniffin’ Sticks Test” revealed normosmic & hyposmic groups. UPDRS, MMSE, MoCA, LEDD.	Hyposmia consistently remained a significant risk factor for FOG development after controlling for age, sex, disease duration, & UPDRS. In the hyposmic group, FOG in 29/79 (36.7%). In the normosmic group, FOG in only 2/29 (7%).	Authors speculate the frontal cognitive deficit is a co-mechanism of action between the olfactory dysfunction and FOG. However, not all PD patients with attention or frontal deficits have FOG. Sampling patients in early-stage might contain a slight risk of including atypical parkinsonian disorders. A limited evidence of cause-and-effect relationship between hyposmia & FOG in retrospective cohort. Testing conducted in ON.	8
Lo et al.^30^	Prospective Cohort	*n* 201, FOG in 41, (20.3%)	Self-report (# 3 FOG-Q)	1.5 years	Smartphone tests in clinic |& at home assessing voice, balance, gait, reaction time, dexterity, rest, & postural tremor. Motor cognitive and functional assessments & questionnaires.	Participants who developed freezing had a longer disease duration and higher MDS-UPDRS part III scores.	Reliance on the accuracy of self-report & subjective questionnaires, with potential recall bias. Limited diagnostic accuracy of PD. Prediction accuracies reported here were obtained using only a single machine-learning algorithm (random forests).	4
Ou et al.^84^	Prospective cohort	*n* 225, FOG in 85, (38%)	Self-report (# 3 FOG-Q)	3 years	Multiple motor & non-motor aspects including depression and anxiety, festination FOG and falls.	Longer disease duration, onset in lower limbs, higher annual changes in UPDRS III & worse visuospatial /executive abilities and presence of festination, falls, and hallucinations were associated with FOG development.	Causal relationship between FOG and PD deterioration cannot be shown. Self-report nature of FOG. No clinical predictors for phenotypes of FOG, lacking OFF / ON testing. Visuospatial/executive item from MoCA may not be comprehensive enough.	8
Prange et al.^85^	Retrospective cohort	*n* 1232, FOG in 128, (10.3%)	MDS-UPDRS items 2.13 and 3.11	Up to 12 years	Seven outcomes from MDS-UPDRS items ≥ 1 motor fluctuations, dyskinesias, postural instability and falls, FOG, impulse control disorders, hallucinations & psychosis, and dementia.	Age at diagnosis > 70 years, bilateral and more symmetrical symptoms at onset determined increased risk for FOG.	FOG risk rose dramatically with disease duration, 10-fold increase in risk intensity 10 years after diagnosis vs. 1 year after diagnosis. Hospital-based recruitment may result in selection bias, although this is mitigated because of systematic evaluation of all complications in an unselected consecutive outpatient cohort. Lack of quantitative description of comorbidities, & details on prescription patterns of antiparkinsonian treatments.	6
Tang et al.^27^	Prospective cohort	*n* 163, FOG in 18 (35%)	N-FOGQ with video & clinical observation	1.5 years	Demographics, four scales of sleep.	During follow-up, (35%) developed FOG; 8 (73%) in the PDSS1 < 6; 10 (24%) in the PDSS1 ≥ 6. Low PDSS and higher LEDD indicate increased risk of developing FOG.	NFOG-Q and PDSS1 are subjective scales. The study was conducted only in ON state.	8
Wang et al.^86^	Retrospective cohort	*n* 183, FOG in 68 (37.2%) By self-report 57, (31%); by clinical observation 32, (17.5%)	MDS-UPDRS items 2.13 and 3.11 Self-report or clinical observation	5 years	Total MDS-UPDRS, multiple motor and non-motor aspects, e.g., MoCA, anxiety, depression, sleep, smell, CSF 123I-FP-CIT SPECT, neuroimaging and genetics.	High PIGD score, fatigue, worse SDMT performance & low levels of CSF Abeta42 were independent risk factors for FOG. Reduction of DAT uptake in the caudate and putamen, and CSF biomarker Abeta42 were predictors of FOG.	Retrospective study with a limited number of subjects. The participants were well educated. Lack of medication states of patients. Severity of FOG was not analyzed. UPDRS II may be less sensitive than the (FOG-Q), underestimated number of freezers. Missing values were deleted and this may introduce selection bias.	6
Xu et al.^26^	Prospective cohort	*n* 967, FOG in 255 (26.4%)	UPDRS-# 14 and New (NFOG-Q)	1 year	Demographics, medical history, family history, PD medication regime, various motor and non-motor features.	Patients with FOG had longer disease duration, greater age and H&Y stage at baseline, lower proportion of Tremor Dominant subtype, and higher proportion in wearing-off, LEDD, usage of L-Dopa and catechol-O-methyl-transferase (COMT) inhibitors, higher scores on UPDRS, PIGD, PDQ-39, NMSS, HDRS-17, PFS, RBDQ-HK, ESS and lower score in PDSS. Patients with FOG had more constipation, RBD, depressive symptoms and excessive daytime sleepiness.	Assessment of FOG was based mainly on outpatient consultation and clinical scales. Follow-up period was relatively short. The patients had large age differences.	6
Zhang et al.^87^	Prospective cohort	*n* 248, FOG in 128, (51.6%)	Report from patient, family member or caregiver & clinical observation	3 years	Anti-parkinsonian medication, medical history, motor phenotype, mood disorders.	FOG after 3 years was associated with: older age (>65 y), living in the countryside, lower education (<9 y), akinetic-rigid phenotype, lower limbs as site of onset, early use of levodopa, higher daily dose of levodopa, not using amantadine/ selegiline/ dopamine receptor agonizts, higher NNMS & HAMD scores at baseline, especially on cognitive and sleep disorders, anxiety & depression.	Estimation of FOG incidence may be affected by recall bias and occurring mostly at home rather than in clinic visit. Results might have been affected by some unpredictable factors, such as the progress of the disease. Drug compliance, anti-Parkinson and antianxiety drugs were different among patients.	6
Zhao et al.^29^	Prospective Cohort	*n* 350, FOG in 132 (37.7%)	NFOG-Q reported by patients, their family members, or caregivers. & clinical observing.	2 years	Demographics, comorbidities, medication usage, modified H & Y stage, disease duration, Motor symptoms, anxious and depressive symptoms, Global cognitive function.	Longer disease duration, higher total levodopa equivalent daily dose, and higher severity of depressive symptoms were the strongest predictors of FOG *For a 100-mg increase in LEDD at baseline, the risk of incident FOG during 2-year increased by 44.0%.	Lack of an independent external validation cohort makes the external generalizability of the model unknown. The interval between follow-up visits among patients was not strictly restricted, so the exact conversion time point cannot be determined. FOG predictors were not discriminated in patients with different medication states. No FOG-specific eliciting tasks have been included in the assessments, which could have improved the objective FOG detection. Patients with DBS surgery were excluded.	6

*FOG-Q* freezing of gait questionnaire, *N-FOGQ* the new FOG questionnaire, *TD* tremor dominant, *PIGD* postural instability gait difficulty, *LEDD* levodopa equivalent daily dos, *UPDRS* unified Parkinson disease rating scale, *LD* Levodopa, *GDS* geriatric depression scale, *PDSS*- Parkinson’s disease sleep scale, *SDMT* symbol digit modalities test, *LID* levodopa induce dyskinesia, *MMSE* Mini mental state Exam, *MoCA* Montreal cognitive assessment, *NA* not available, *DAT* striatal dopamine transporter, *NPI* neuropsychiatric inventory, *NPS* neuropsychiatric symptoms, *GABS* gait and balance scale, *NMS* non-motor symptoms, *HAMD*/*HDRS* Hamilton depression rating scale, *HAMA* Hamilton anxiety rating scale, *DBS* deep brain stimulation, *PFS* Parkinson’s fatigue scale, *RBDQ-HK* rapid eye movement sleep behavior disorder questionnaire-Hong Kong, *ESS* Epworth sleepiness scale, *CSF* cerebrospinal fluid, *COMT* Catechol-O-methyl-transferase, *TMT* trail making test, *H&Y* Hoehn and Yahr stage.

**Table 2:** Brain imaging and other modalities and their association with the future development of FOG.

Authors, year	Study design	Sample size (*n* total, % who developed FOG)	FOG ascertainment	Duration of Follow-up (years)	Exposure Predictors	Main findings	Remarks and limitations	Total quality score
Chung, Lee et al.^34^	Retrospective cohort	*n* 268, FOG in 52 (19.4%)	Self-report	5.9 ± 1.6, (WMH-) 5.0 ± 1.4, (WMH + )	WMH visual rating scale scores	After adjusting for age, sex, DAT, and LED, WMH + group showed a higher risk of developing FOG (HR, 3.29; 95%CI, 1.79–6.05; *p* < 0.001) than the PD-WMH- group. (PD-WMH- *n* = 198; FOG in 27 (14%); in PD-WMH + *n* = 70; FOG in 25 (36%).	Ischemia classification system is not very accurate. Lack of DTI data. FOG was not assessed objectively. Individual variability in the intensity of vascular risk factors.	7
Chung, Yoo et al.^52^	Retrospective cohort	*n* 139, FOG in 38 (27.3%)	Self-report & clinical observation	At least 3 years	Age at disease onset, (DAT) availability, motor and non-motor symptoms.	Older-onset PD group had higher risk for FOG than the younger-onset group.	Patients’ subdivision into groups is somewhat arbitrary. FP-CIT PET may not be ideal surrogate marker for nigrostriatal dopaminergic degeneration. FOG was not assessed using objective gait measures. Atypical parkinsonism cases might be misdiagnosed as idiopathic PD. Due to the lack of follow up data clinical progression was assessed based on the longitudinal changes in LED, which might not accurately reflect the motor status.	8
Chung et al.^88^	Prospective cohort	*n* 205, FOG in 51 (24.9%)	Self-report & Clinical observation	6.31 ± 1.59 years	Motor reserve	Greater motor reserve estimates, as determined by initial motor deficits and striatal dopamine depletion, were associated with a lower risk for FOG.	Motor-symptom laterality and interhemispheric symmetry of dopamine loss were not taken into account. FOG retrospective determination was based on medical records, so mild cases can be missed. Disease duration could be measured with error. There were no follow-up assessments for motor deficits or striatal DAT depletion. The longitudinal changes in LED may not be the ideal measures for disease progression. DAT availability may not purely reflect the nigrostriatal dopaminergic denervation.	8
Chung, Yoo, Shin et al.^89^	Retrospective cohort	*n* 248, FOG in 64 (25.8%)	Self-report & clinical observation	6.00 ± 1.85 years in PDEPVS−Group 6.35 ± 2.13 years in PD-EPVS + group	High number (>10) of enlarged perivascular spaces in the basal ganglia (EPVS + ).	FOG was higher in the PD EPVS + group; FOG in 35% vs. 20.5% in the EPVS- group.	Visual scoring of BGPVS only provided qualitative estimates of the PVS extent. Enlarged BG-PVS were only assessed at the baseline evaluation. Minimal FOG might not be detected early. Direct measures of motor deficits were not collected in a longitudinal manner.	8
D’Cruz et al.^90^	Prospective cohort	2 studies: MRI- *n* 45, FOG in 9 (20%) f-MRI- n 41, FOG in 7 (17%)	Self-report	2 years	Structural MRI. Resting state fMRI Connectivity between sub-thalamic nuclei/cortical areas.	Converters had higher LEDD and higher MDS-UPDRS part I and total scores. Freezers and converters showed significant local shape inflations compared to non-converters in the right and left thalamus. Gender, LEDD, and cluster from left thalamus were significant predictors of conversion to FOG.	Low conversion rate- only 20%. Differences in disease stage. Results may relate to a particular FOG phenotype.	7
Djaldetti et al.^35^	Retrospective cohort	*n* 41, FOG in 15 (34.8%)	UPDRS part II	2 years	DAT binding in striatum, age at symptom onset, age at DaTSCAN, disease type, UPDRS part III score during “on” at baseline and end of follow-up, duration of levodopa treatment, dosage of levodopa at end of follow-up.	DAT uptake values were significantly lower in the putamen and striatum in patients with FOG.	Relatively small number of cases. Retrospective design. Cognitive tests and assessment for depression, anxiety, and other behavioral features were not administered. Most of the patients were already under treatment at the time of imaging.	8
Kim et al.^36^	Retrospective cohort	*n* 390, FOG in 143 (36.7%)	MDS-UPDRS	4 years	Baseline dopamine transporter (DAT) uptake in caudate nucleus and putamen.	DAT uptakes in the caudate nucleus and putamen predicted the development of FOG. Severe reduction of DAT uptake in caudate nucleus and putamen had a higher incidence of FOG than mild and moderate reduction. Male sex, higher PIGD score, and lower MoCA score were also significant predictors of FOG.	Initial DAT scan may not reflect a precise prediction of disease progression.	8
Kim et al.^37^	Prospective cohort	*n* 339, FOG in 142, (42%)	MDS-UPDRS	6 years	APOE ε4, motor, mood and cognitive symptoms	FOG in 45 out of 88 (51%) with positive APOE ε4 allele vs. 97 out of 251 (39%) in the negative group. CSF Aβ42, male sex, age at onset, MDS-UPDRS motor score, PIGD score, MoCA score, GDS score, and DAT uptakes in the caudate nucleus and putamen were significantly related to FOG.	Results may be different if amyloid levels are measured by PET imaging rather by CSF Aβ42. FOG may be underestimated after starting dopaminergic medications.	7
Kim, Lee et al.^39^	Prospective cohort	*n* 393, FOG in 136 (35%)	MDS-UPDRS	4 years	CSF parameters, Demographic and clinical data, motor and non-motor symptoms, DAT imaging.	CSF Aβ42 was associated with FOG. Male sex, MDS-UDPRS motor, PIGD score, MoCA score, and caudate DAT uptake were also significantly predictive of FOG. *The combined model integrating the PIGD score, caudate DAT uptake, and CSF Aβ42 achieved a better discriminative ability than any factor alone. Cumulative incidence of FOG: 16.5%, 21.0%, 28.2%, and 36.7% at the 1, 2, 3, and 4-year follow up, respectively.	The data does not measure the extent of amyloid pathology. Relatively short follow-up period. FOG might be misestimated through MDS-UPDRS. FOG may be underestimated after dopaminergic drugs initiation.	5
Kim and Jeon, ^91^	Retrospective cohort	*n* 361, FOG in 189 incidence rates of 22, 37, 48, and 63% at the 2-, 4-, 6- and 8-year follow-ups, respectively	MDS-UPDRS items 2.13 and 3.11	8 years	Nfl, demographics, motor and non-motor symptoms, DAT scan.	NfL levels are a novel risk factor for FOG.	FOG might be misestimated through self-report. FOG could not be differentiated according to medication states.	5
Li et al.^92^	Prospective cohort	*n* 40, FOG in 20, (50%) matched to no-FOG	NFOG-Q item 1	5 years	Topological organization of whole brain functional networks (fMRI).	The PD with FOG group exhibited decreased nodal centrality in the left middle frontal gyrus (MFG).	Relatively small sample size. FOG was self-reported rather than objectively measured. A small part of patients received optimized anti-PD medication at baseline, so FOG during the follow-up may have been interfered by pharmacological effects.	8
Li et al.^93^	Retrospective cohort	*n* 158, FOG in 66 (42%)	UPDRS items 2.13 OR item 3.11	5 years	Demographics, clinical and laboratory characteristics, MRI T1 imaging.	T1-weighted imaging predicts FOG. PD patients who developed FOG had decreased olfactory function, depression, a more severe disease degree, dysfunction of daily living and movement, postural instability, and gait difficulty at baseline. Men are more prone to FOG.	Only T1 MRI analysis was performed. A limited amount of clinical data. Different machine-learning methods might change the results in diverse cohorts. Participants were mainly from European and American populations.	5
Dadar et al.^38^	Prospective cohort	*n* 423, FOG in 177 (42%)	MDS-UPDRS items 2.13 and 3.11	5 years	T1w MRI: DBM for SN Atrophy. Segmentation of WMH. Putamen and caudate DAT levels. Cerebrospinal fluid (CSF) amyloid β.	WMH load mediated the impact of amyloid β on future FOG.	No consistent follow-up information available for all participants. Lack of gait characteristics. Limited spectrum of disease.	8
Sarasso et al.^94^	Prospective cohort	*n* 22, FOG in 11 (50%)	Observation by neurologist & FOG Questionnaire	2 years	Demographics, motor and non-motor symptoms, MRI biomarkers.	FOG-converters showed greater PIGD, more severe limb/axial rigidity and more frequent dyskinesia. Prediction model including dyskinesia, PIGD scores and parietal clustering coefficient were the best predictor of FOG conversion.	Study samples were small and matched for UPDRS-III. There was no longitudinal MRI data in healthy subjects that could control for the aging effects. 1.5 T MRI scanner was used, which has lower spatial resolution and BOLD signal to noise ratio resolution. PD patients were evaluated in ON status. A longer follow-up might improve the possibility to monitor FOG conversion. Prediction model might suffer from overfitting of data due to small sample size.	8
Wieler et al.^95^	Prospective cohort	*n* 19, FOG in 7 (37%)	UPDRS II	3 years	Nigral iron content (MRI), motor and non-motor symptoms.	FOG was associated with motor impairment at baseline (UPDRS III scores), time to complete a 14 m walk, Berg Balance scale and timed up and go, more rapid deterioration in motor function, higher levodopa equivalent dose at baseline and increased iron content in lateral SN pars compacta.	FOG might be underestimated since it is mostly self-reported. There was no differentiation between “ON” freezing and “OFF” freezing. Small sample.	6

*SN* substantia nigra, *BG* basal ganglia, *DBM* deformation-based morphometry, *WMH* white matter hyperintensities, *DTI* diffusion tensor imaging, *EPVS* enlarged perivascular spaces, *NfL* neurofilament light chain. For additional abbreviations please also see Table S1 in the supplement.

### Quality assessment

The overall quality score for each study is presented in Tables 1, 2 (rightmost column). Quality scores ranged between 4 and 8 stars with a median [quartiles] of 6.5 [6–8]. Of the 38 studies, 29 were rated as high quality (76.3%, see Supplementary Table 2 for the full quality assessment).

### Systematic review

Most of the studies evaluated a large number of potential motor and non-motor predictors using different scales, procedures, and tests that assessed several disease features (e.g., measures reflecting disease severity, motor function, cognitive function, anxiety, sleep, depression, hyposmia, and anti-Parkinsonian medications) (Table 1). Of those, several factors were associated with the future development of FOG. For example, Zhao et al.^29^ (*n* = 350, 37.7% developed FOG) found that longer disease duration, higher levels of levodopa equivalent daily dose (LEDD), and a more depressive state at baseline were significantly associated FOG incidence. A 100-mg relatively higher level of LEDD at baseline was associated with a 44% increased ‘2-year risk’ of FOG. Not surprisingly, all studies did not agree. For example, in the prospective study by Chung, Yoo, Lee, et al.^28^ in 257 patients who were followed for at least 2 years, none of the predictors identified by Zhao et al.^29^ were reported as being associated with FOG incidence.

Imaging and other possible predictors were also explored (Table 2). White matter hyperintensities increased the risk of developing FOG more than 3-fold (HR:3.29: CI:1.79–6.05), even after controlling for age, sex, DAT uptake, and LEDD^31^. Lower DAT uptake values were significantly related to a higher FOG incidence^32,33^. A positive APOE ε4 allele was moderately associated with a higher risk of developing FOG^34^. Lower levels of CSF Aβ42 (inversely related to brain amyloid load) were associated with an increased risk of FOG in two studies^35,36^. Likely because different studies examined disparate possible risk factors, agreement across these studies was often lacking.

### Meta-analysis

**Fig. 2:**
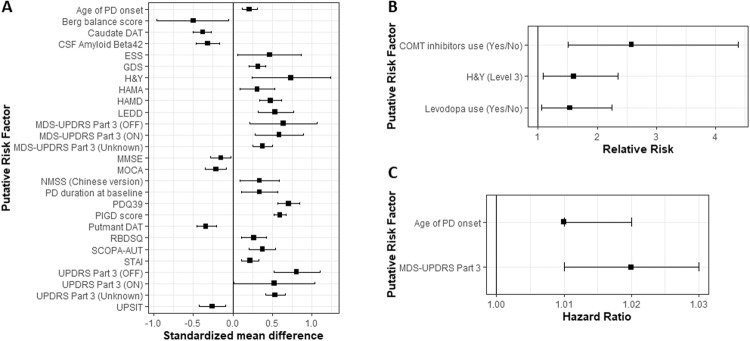
Summary of significant risk factors for incident FOG according to the type of data available with effect size and 95% confidence interval of random effects models. In each panel, the factors are organized in alphabetical order. **A** Standardized mean difference; (**B**) Relative risk; **C** Hazard ratio. A list of all of the abbreviations used can be found in the Supplementary Table 1.

**Fig. 3:**
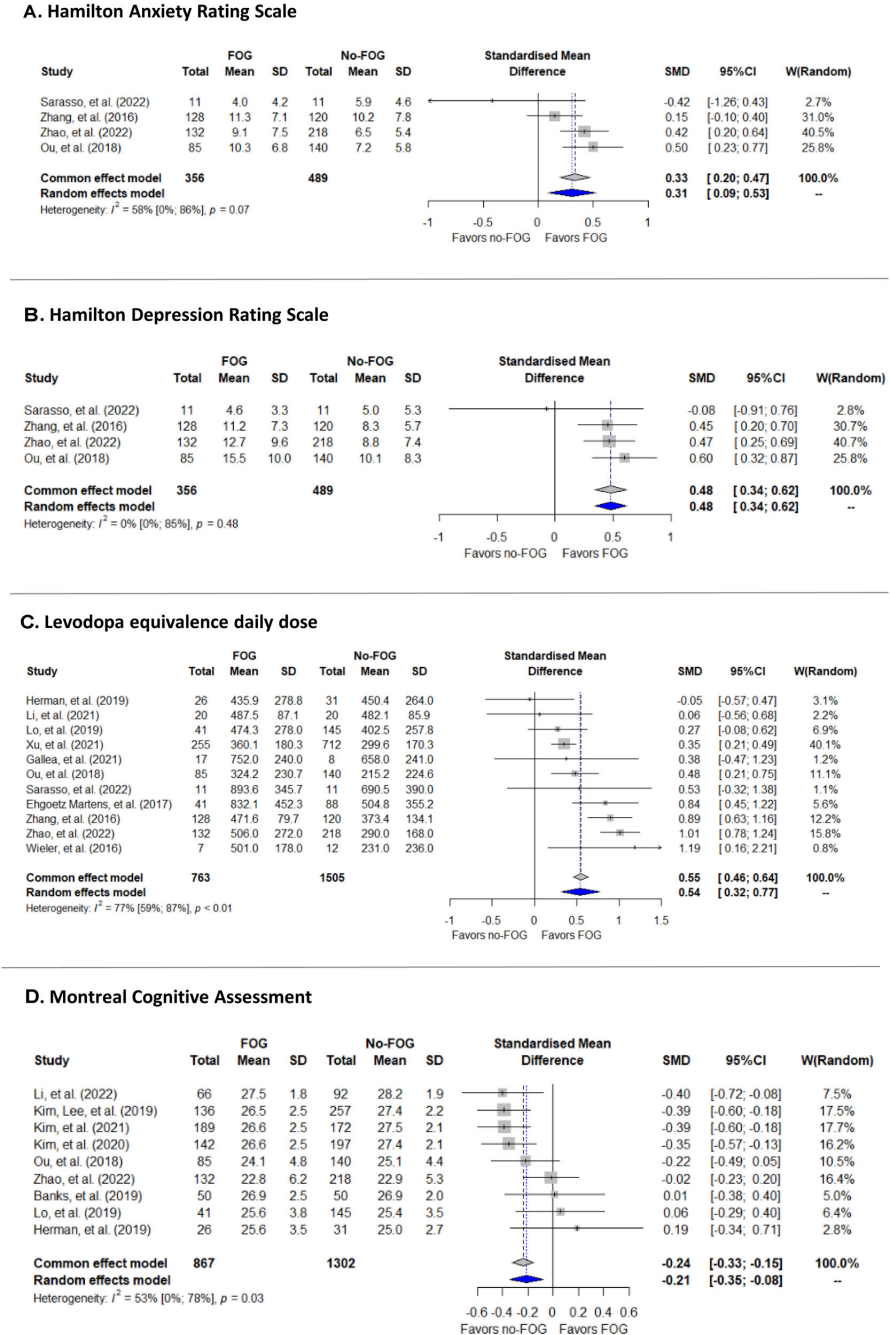
Forest plots of selected significant predictors of the future development of FOG. **A** represents one of the anxiety predictors; (**B**) represents one of the depression predictors; (**C**) represents the use of medication predictors; (**D**) represents one of the cognitive domain predictors.

Thirty-five studies were included in the meta-analysis (8,973 participants). Among those, 2175 people (24.2%) developed FOG by the end of follow-up (mean follow-up: 4.1 ± 2.7 years) (Table 3, note that variables that were reported only in two studies are presented in Supplementary Table 3). Non-modifiable predictors included older age at disease onset, longer disease duration, and lower DAT uptakes (both in the caudate and putamen). Figure 2 summarizes the significant predictors. Figure 3 presents the Forest plots of selected significant predictors of FOG.

**Table 3:** Main meta-analysis results presented by categories.

Putative risk factor		*N*	*N*		Statistic	Random effects	Heterogeneity	Publication bias
		Studies	FOG	No-FOG		Effect size (95%CI)	LOU effect size (95%CI), I^2^	
Demographics and general characteristics	Age at baseline	10	785	1596	SMD	0.11 (−0.07, 0.29)	Substantial	No
	Age of PD onset	8	899	1607	SMD	0.21 (0.12, 0.31)^a^	Small	No
		6	490	2182	HR	1.01 (1.01, 1.02)^a^	Considerable 1.01 (1.01, 1.02) ^a^, I_2_ = 0%	No
	Age of PD onset (early PD = 1)	4	450	949	RR	0.69 (0.35, 1.34)	Substantial	No
	Sex (Female = 1)	21	1571	2949	RR	0.89 (0.78, 1.03)	Substantial	No
		13	847	4434	HR	1.00 (0.93, 1.08)	Small	No
	Education (Years)	7	265	356	SMD	−0.11 (−0.27, 0.06)	Small	No
	PD duration at baseline	14	1117	2037	SMD	0.34 (0.11, 0.57)^a^	Considerable 0.28 (0.06, 0.50)^a^, I_2_ = 83%	No
Motor features	Berg balance test score	3	165	261	SMD	−0.50 (−0.96, −0.05)^a^	Substantial	Yes
	Hoehn and Yahr	5	199	271	SMD	0.74 (0.24, 1.24)^a^	Considerable 0.48 (0.22, 0.73)^a^, I_2_ = 0%	No
	MDS-UPDRS Part 3 (OFF)	3	253	368	SMD	0.64 (0.22, 1.07)^a^	Considerable 0.37 (0.17, 0.57), I_2_ = 0%	No
	MDS-UPDRS Part 3 (ON)	3	78	130	SMD	0.59 (0.28, 0.90)^a^	Small	No
	MDS-UPDRS Part 3 (Unknown)	4	498	807	SMD	0.38 (0.25, 0.50)^a^	Small	No
	UPDRS Part 3 (OFF)	3	90	112	SMD	0.81 (0.52, 1.11)^a^	Small	No
	PD motor subtype (intermediate)	3	208	279	RR	0.66 (0.19, 2.35)	Considerable 0.95 (0.13, 6.77), I_2_ = 54%	No
	PD motor subtype (PIGD)	6	504	1048	RR	1.53 (0.86, 2.74)	Considerable 1.20 (0.84, 1.71), I_2_ = 57%	No
	PD motor subtype (tremor dominant)	6	504	1048	RR	0.82 (0.68, 1.00)	Moderate	No
	PIGD score	7	931	1659	SMD	0.60 (0.52, 0.68)^a^	Small	No
	Tremor score	6	920	1648	SMD	0.01 (−0.09, 0.11)	Moderate	No
Non-motor features	Geriatric Depression Scale	6	609	799	SMD	0.32 (0.21, 0.42)^a^	Small	Yes
	HAMA	4	356	489	SMD	0.31 (0.09, 0.53)^a^	Substantial	No
	HAMD	4	356	489	SMD	0.48 (0.34, 0.62)^a^	Small	No
	NMS-Quest	3	165	162	SMD	0.85 (−0.31, 2.01)	Considerable 0.33 (−0.34, 0.99), I_2_ = 47%	No
	PDQ39	3	288	755	SMD	0.71 (0.57, 0.85)^a^	Small	No
	REM Sleep Behavior Disorder Questionnaire	4	263	410	SMD	0.27 (0.11, 0.43)^a^	Small	No
	SCOPA-AUT	3	228	380	SMD	0.38 (0.21, 0.54)^a^	Small	No
	State-Trait Anxiety Inventory	5	583	768	SMD	0.22 (0.11, 0.33)^a^	Small	No
Cognitive function	MMSE	4	329	843	SMD	−0.15 (−0.28, −0.02)^a^	Small	No
	MOCA	9	867	1302	SMD	−0.21 (−0.35, −0.08)^a^	Substantial	No
Medication	Amantadine use (Yes/No)	4	475	984	RR	0.76 (0.33, 1.76)	Considerable 1.23 (0.82, 1.84), I_2_ = 0%	No
	Dopamine agonist use (Yes/No)	4	475	984	RR	0.59 (0.22, 1.54)	Considerable 1.07 (0.95, 1.20) I_2_ = 0%	No
	LEDD	11	763	1505	SMD	0.54 (0.32, 0.77)^a^	Considerable 0.48 (0.26, 0.69) ^a^, I_2_ = 64%	No
		4	199	696	HR	1.00 (1.00, 1.00)	Considerable 1.00 (1.00, 1.00), I_2_ = 30%	No
	Levodopa use (Yes/No)	4	475	984	RR	1.54 (1.06, 2.25)^a^	Considerable 1.23 (0.99, 1.52), I_2_ = 68%	No
Imaging	Caudate DAT	4	535	741	SMD	−0.38 (−0.50, −0.27)^a^	Small	No
	Putman DAT	3	467	626	SMD	−0.34 (−0.46, −0.21)^a^	Small	No
		7	312	1441	HR	0.86 (0.75, 0.99)^a^	Substantial	Yes

^a^*p* < 0.05.

**Fig. 4:**
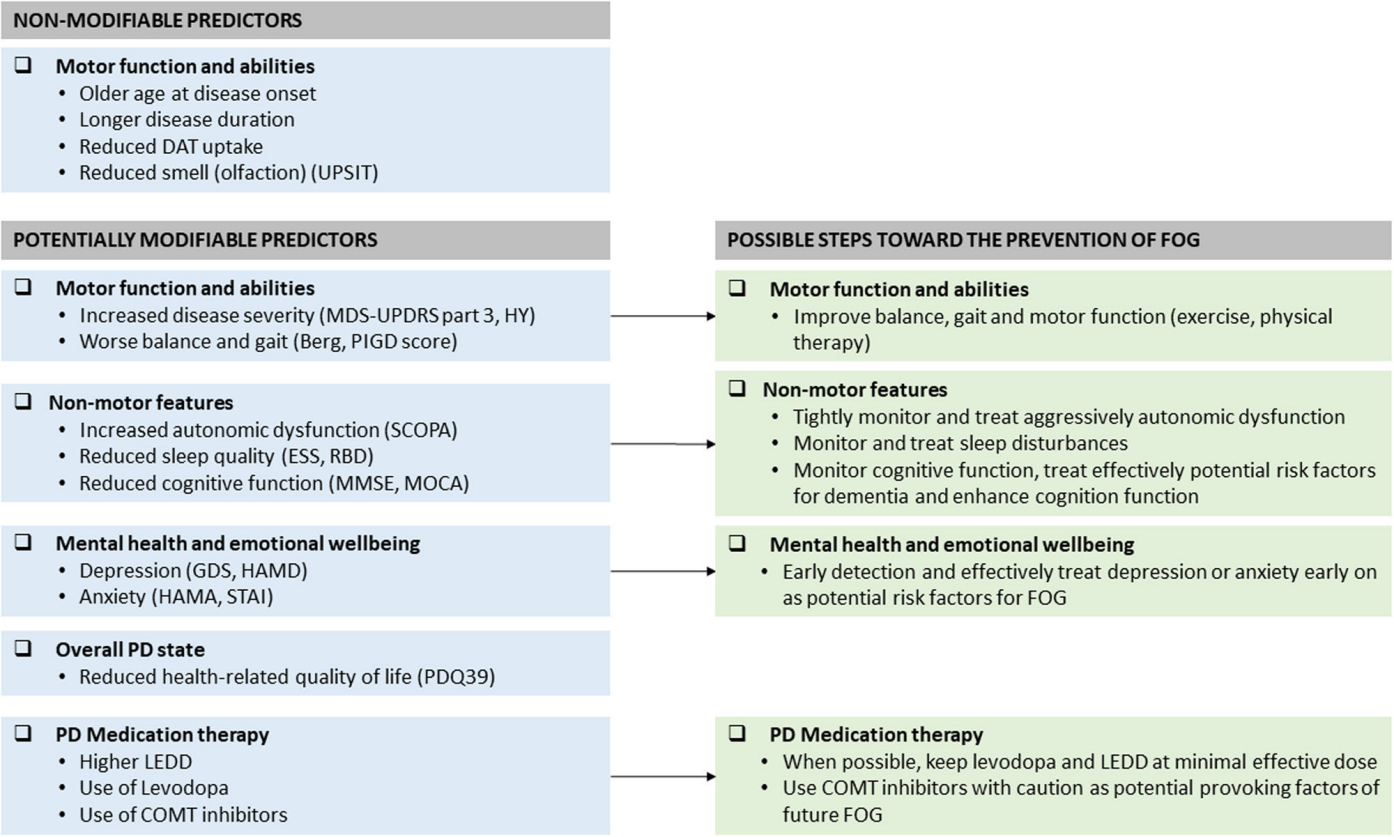
Summary of the significant predictors of FOG incidence, grouped with respect to those that are putatively modifiable and those that are not. When possible, we recommend keeping levodopa and LEDD at minimal effective doses. When anti-parkinsonian medications are prescribed, the patient’s response should be carefully monitored. A list of all of the abbreviations used can be found in the Supplementary Table 1.

Several modifiable PD features were also identified (Fig. 3). For example: (1) higher baseline levels of depression and/or anxiety (Fig. 2A: GDS, HAMA, HAND, STAI and Fig. 3A, B); (2) poorer baseline motor function, gait, and balance (Fig. 2A: Berg balance test, H&Y, MDS-UPDRS part3, UPDRS part 3, PIGD score; Fig. 2B: H&Y level 3, Fig. 2C: MDS-UPDRS part 3); (3) higher LEDD at baseline (Fig. 3C); (4) use of Levodopa and COMT inhibitors (Fig. 2B); (5) a worse cognitive state at baseline increased the risk for the future development of FOG (Fig. 2A: MOCA, MMSE and Fig. 3D); and (6) non-motor features such as autonomic dysfunction, reduced sleep quality (measured by ESS and RBDSQ, Fig. 2A), olfactory deficiency (measured by UPSIT, Fig. 2A) and worse PDQ39 scores (Fig. 2A), also predicted FOG development.

The results of the multivariable analysis were similar to those of the main analysis (Table 4). Even when simultaneously taking into account and adjusting for age at disease onset, disease duration, depression, cognitive function, motor severity, and LEDD levels, all of these factors were still significantly associated with FOG incidence. Most results were similar when MOCA was replaced by MMSE and when STAI was replaced by HAMA.

**Table 4:** Multivariable analysis results (vs. main analysis).

	Main analysis SMD (95%CI)	Multivariable SMD (95%CI)
Age at PD onset	0.21 (0.12, 0.31)^a^	0.27 (0.14, 0.4)^a^
PD duration	0.34 (0.11, 0.57)^a^	0.38 (0.26, 0.5)^a^
Geriatric Depression Scale	0.32 (0.21, 0.42) ^a^	0.49 (0.34, 0.64)^a^
LEDD	0.54 (0.32, 0.77)^a^	0.48 (0.35, 0.62)^a^
MDS-UPDRS part 3^b^	0.50 (0.34, 0.65)^a^	0.51 (0.38, 0.65)^a^
MOCA	−0.21 (−0.35, −0.08)^a^	−0.17 (−0.31, −0.04)^a^
State-Trait Anxiety Inventory	0.22 (0.11, 0.33)^a^	0.4 (0.25, 0.55)^a^

^a^*p* < 0.05.

^b^We included all MDS-UPDRS part 3 (OFF, ON and unknown).

### Heterogeneity

Overall, the majority of analyses had small to moderate heterogeneity (36/62, 58.1%). Considerable heterogeneity was assessed in 13 risk factors (20.9% of analyses). The leave one out (LOU) analysis was calculated for 12/13 analyses (‘hyposmia’ had only two studies). Out of the 12 LOU analyses, the heterogeneity of 7 features was reduced to small/moderate, 4 to substantial (levodopa use, LEDD continuous and PIGD and intimidate PD subtypes), and only 1 (PD duration) remained considerable. Overall, the effect sizes did not differ from those in the main analysis.

### Publication bias

Out of 62 analyses, Egger’s intercept calculation was applicable to 32. Of those, only 3 analyses had a high risk of publication bias (i.e., Berg balance score, Putamen DAT, and H&Y [Level 1, RR]). The intercepts and p-values are presented in Supplementary Table 4.

### Subgroup analysis

Four subgroup analyses were performed: (1) by study quality score (≥6; 26/35 studies, median [IQR] = 10 [9–10.25]), (2) by study design (prospective: 21/35 studies), (3) by follow-up period (≥2 years: 30/35 studies, median [IQR] = 3.5 [2–5]), and (4) by PD duration at baseline (<2 years: 19/35 studies, median [IQR] = 2 [0.7–6]). Several modifiable outcomes that were identified as statistically significant predictors in the main analyses were included in the subgroup analyses: motor and disease severity, LEDD, depression and anxiety, cognitive state, and quality of life. Generally, the results did not differ from those shown in the main analysis (Supplementary Table 5). However, MOCA, MMSE, and MDS-UPDRS part 3 (ON) lost their significance when assessing studies with a follow-up period of more than 2 years. In addition, levodopa use was no longer a significant risk factor in studies including patients with longer disease duration at baseline (over 2 years PD duration, RR: 1.17 [0.98–1.40]). Nonetheless, the risk for developing FOG in newly diagnosed patients using levodopa at baseline was greater compared to the main analysis (RR new PD: 2.3 [1.77–2.97], RR main analysis: 1.54 [1.06, 2.25]).

## Discussion

The present systematic review and meta-analysis generate new insights into risk factors that are associated with the incidence of FOG in patients with PD. Multiple factors were identified that were different in people with people who later developed PD, compared to those who did not, raising the possibility of prediction. Interestingly, some of these risk factors are modifiable, suggesting that perhaps even prevention can also be considered. These findings carry important implications for the clinical management of PD patients, enhancing our understanding of the underlying pathological mechanisms contributing to the development of FOG, as well as offering potential avenues for predicting, delaying, and perhaps even preventing FOG.

The inter-relationships between FOG incidence, disease duration, and severity are complex and challenging to disentangle. The meta-analysis shed some light on these relationships. According to previous reports, disease duration, and disease severity (e.g., scores on MDS-UPDRS part III) increase the risk of developing FOG^1,37–40^. Although less prevalent, FOG may also occur early in the disease^1,36–40^. In line with these reports, the meta-analysis found that an increase in disease duration was associated with an increased risk for FOG development, even after adjusting for motor and non-motor baseline symptoms and LEDD (Table 4), supporting the idea that FOG risk increases as disease duration and severity increases. However, in the subgroup analysis (Supplementary Table 5), we found that even in patients with less than 2 years of PD, a relative increase in disease severity (i.e., MDS-UPDRS part III), anxiety, depression, balance impairment, cognitive impairment, and higher levels of LEDD still predicted the development of FOG. This suggests that already among people with relatively short disease duration, certain factors exist that are associated with an increased risk of developing FOG in the future. Furthermore, in the multivariable analysis, the severity of those symptoms was independently associated with an increased risk of developing FOG (Table 4). Exclusion of patients with DBS was not specified as an exclusion criterion, but since we excluded patients with FOG at baseline, the included studies had very few advanced PD patients; the median disease duration at baseline (study entry) was only 1.8 years [IQR: 0.125–4.25 y]. Nevertheless, one should bear in mind that disease duration cannot be a proxy of disease severity, for example in the case of ‘malignant form’ which generally accounts for 9–16% of cases of PD or even atypical parkinsonism that was diagnosed by mistake as PD (less than 0.1% of cases).

The findings of this review support the idea that the pathological processes contributing to the development of FOG may be present years before FOG becomes clinically evident, at least in some subpopulations. Consistent with this notion, in several of the reviewed studies that matched the subjects at baseline with respect to disease duration and the severity of motor symptoms, non-motor factors differed in individuals who later developed FOG and those who did not during 5–10 years of follow-up. The role of non-motor symptoms in FOG is not surprising. Indeed, affect, depression and anxiety have been shown to be increased in people with FOG, compared to people with PD who do not have FOG^41,42^. Extending that idea, the present analyses support the position that these alterations in mental health not only are related to and may trigger specific FOG episodes, among people who experience FOG, but in addition, these non-motor alterations may be in the causal pathway and lead to the future development of FOG.

The complexity of the relationship between disease severity and duration becomes more entangled when considering the debated contribution of anti-parkinsonian medications to the development of FOG^43,44^. Regarding levodopa usage, there are arguments both in support of and against its role in increasing the risk of FOG. The current analysis, like several past studies^43–45^, shows that patients prescribed levodopa had a higher risk of developing FOG, compared to those who were not (RR: 1.54). Some have explained that levodopa likely does not enhance nigral neurodegeneration and its potential to damage dopamine cells is minimal^46^. On the other hand, although LEDD might just be a reflection of disease severity and not a risk factor by itself, in the multivariate analysis, we found that a higher dose of LEDD was associated with increased risk for FOG, even when adjusting for other potential confounders. Furthermore, Koehler and colleagues^47^ speculate that high-frequency oscillatory features of FOG are probably induced by levodopa. Recently, Jansen et al.^48^ introduced the idea that LEDD can exacerbate the risk of developing FOG. Strikingly, they found that FOG was 6 times more common in a levodopa-treated cohort than in a naive cohort. Conversely, Gilat et al.^45^ argue that FOG can manifest before levodopa intake (e.g., it is present in 5–17% of naïve patients). This discrepancy highlights the need for longitudinal studies to determine how the interplay between evolving pathology and dopaminergic therapy affects the development of FOG. Still, it is important to keep in mind that LEDD was associated with FOG development. To further investigate these relationships and dependencies, there is a need for future pre-specified studies. Since isolating the effects of medication from disease severity can be challenging, caution should be exercised when interpreting and acting on these findings. Moreover, there is a need for future work on the association between FOG and specific medications, e.g., benzodiazepines, SSRIs/SNRIs, in addition to levodopa.

In addition, the use of COMT inhibitors was associated with a significant increase in the risk of developing FOG, as evidenced by an RR of 2.58 (based on two studies). Furthermore, in line with the existing debate^43^, and despite what was found in previous randomized controlled studies^5^, the present analysis showed a non-significant result for the association between dopamine agonist therapy and FOG incidence. Moreover, while previous research has shown that MAO-B inhibitors have been associated with a decreased risk of developing FOG^5^, the current analysis observed a non-significant increased risk. Similarly, the use of anticholinergic medications was not significantly associated with FOG incidence (recall Supplementary material). These findings should be considered when prescribing PD medications.

Imaging studies have described multiple pathways and brain regions that are associated with FOG in cross-sectional studies^32,49,50^. Here, we found that alterations in the nigro-striatal pathway were related to the development of FOG^33,49^. This pathway, involving the connections between the substantia nigra and the striatum, plays a crucial role in motor control. However, the behavioral findings suggest that other neural circuits, possibly related to limbic and frontal networks, are also important. These circuits are associated with emotional processing and executive functions and are also involved in gait control and navigation. For instance, the current meta-analysis identified multiple non-motor features such as depression, anxiety, poor cognitive state, and sleep disturbances that were associated with an increased risk of developing FOG in the future. It is not yet clear from this analysis if all of these factors are markers that just predict future FOG development or if they also contribute and play a role in the causal chain. Nonetheless, as noted above, given that non-motor changes like greater depressive symptoms and anxiety have already been shown to be increased in people with PD who have freezing, compared to those without^8,41,51^, perhaps these factors also are causally involved in the future development of FOG, as postulated in a recent model that distinguished between FOG-triggers and longitudinal factors that set the stage for the future development of FOG^8^.

A recent study reported that mutations in the *GBA* gene were associated with increased FOG incidence^52^. This paper was not included in the present review and the meta-analysis because of its publication date. Nonetheless, it highlights the potential role of genetics in evaluating and understanding FOG risk. We also note that that a positive APOE ε4 allele and lower levels of CSF Aβ42 were associated with an increased risk of FOG^34–36^. It was suggested that the allele directly impacts the development of α-synuclein pathology and regulates α-synuclein pathology independent of its established effects on Aβ and tau in mouse models. Thus, it is possible that APOE ε4 carriers have higher quantities of tau and α--synuclein pathology and more severe white matter hyperintensities than APOE ε4 non-carriers, which may affect the neural circuitry associated with FOG. Moreover, the APOE ε4 allele is thought to exert a harmful effect on glucose metabolism and microglial homeostasis. Considering previous findings, these changes may contribute to a faster progression of PD, including cognitive impairment, that was found to predict FOG. Nevertheless, the association of such changes due to the APOE ε4 allele with FOG remains unclear and needs confirmation and further investigation.

Another possibility to consider is that perhaps risk factors that are specific to FOG might be identified, rather than working with thresholds and the degree of symptoms that are common among people with PD (e.g., depression level). Developing a FOG-specific composite index that incorporates multiple risk factors, including depressive symptoms, anxiety, motor function, DAT in the caudate and putamen, other imaging findings, and perhaps genetics, for example, could be a valuable approach, analogous to the likelihood ratio used to identify prodromal PD. This index could provide a comprehensive assessment and help to stratify patients based on their individual risk levels. Initial attempts at developing such composite scores have shown promise, particularly when combining imaging with other measures^36,53^. Considering the low ability to differentiate posterior putamen, ventral putamen and caudate uptake, it would be interesting in the future to explore DAT uptake levels at the different regions of the striatum and how they might correlate with FOG. Additional, prospective observational studies are needed to validate and refine composite indices for FOG risk, both with and without the inclusion of relatively expensive tests like imaging or electroencephalography.

Early identification of people with PD with a relatively increased risk of developing FOG may inform and lead to early interventions to reduce FOG and its devastating consequences in a sub-group of patients with a high FOG risk. Hence, the present findings contribute to the emerging concept of precision medicine for the treatment of PD. Personalized medicine aims to tailor medical interventions to individual patients based on their specific characteristics, including genetic, environmental, and clinical factors^54–57^. By identifying unique risk factors, other associated factors, and underlying pathological processes associated with FOG development, clinicians can potentially adopt a precision medicine approach to the treatment of FOG. This could enable and inform a proactive stance in managing FOG, aiming to prevent or delay its emergence rather than only addressing it reactively only after FOG becomes manifest.

One potential preventive approach that should be further explored could involve early treatment of non-motor risk factors such as anxiety, depression, and executive function deficits^58–61^, keeping lower dosages of daily levodopa when possible, and focusing on interventions to improve gait early in the disease course. Addressing these modifiable non-motor symptoms early on may help alleviate their impact on gait, and reduce the risk of developing FOG. Exercise and physical activity, as well as behavioral, psychological, and pharmacological interventions, can partially alleviate these symptoms^15,43,61–64^. Relatively long-term prospective intervention studies are required to evaluate these important possibilities. Nonetheless, in the meantime, aerobic and multimodal exercise, for example, have minimal negative consequences and multiple positive benefits^65–67^ - irrespective of FOG - and may merit consideration for altering the natural course of the development of FOG, even before randomized controlled trial evidence is obtained.

These possibilities emphasize the importance of a comprehensive assessment that includes both motor and non-motor symptoms in PD management. Nevertheless, many of the identified risk factors are common in PD and aging in general, making it crucial to define clear thresholds to identify when these factors become significant contributors to FOG development e.g., at what level does anxiety become a risk factor for FOG or does that occur only if other factors are also present? (as in the composite risk indices discussed above). FOG poses a significant risk for falls and loss of independence in individuals with PD. Therefore, it is important to explore ways to delay or prevent its development. While there is no single “magic bullet” to fully eliminate the troublesome symptoms associated with FOG, a multi-modal therapeutic approach that addresses the identified risk factors - both motor and non-motor - may prove effective in reducing the risk of FOG development.

This extant literature and the present meta-analyses have several limitations. For example, three studies were excluded from the meta-analysis due to a lack of information that could be integrated with other publications or the absence of the numbers of freezers and non-freezers. Many of the studies recruited subjects at different time points along the natural history of the disease. Ideally, a natural history study starting at disease diagnosis, in untreated patients, or even earlier, could help to clarify the discrepancies between studies. Nonetheless, the subgroup analyses partly addressed this issue. The difference in how FOG was measured also may have contributed to inconsistencies across the studies. About half of the studies used self-report to identify FOG rather than using objective measures. Indeed, in a study among 9072 patients with PD, 51% had FOG based on the NFOG-Q, compared to only 23% based on the UPDRS^40^, highlighting the important role of the method of determining FOG. Similarly, the absence of a detailed description of the PD phenotype, tremor-predominant, PIGD, mild-motor, intermediate, diffuse-malignant subtypes^68^ and report of which classification system was used may have impacted the findings. Work on outcome measurement is needed to address these issues. The duration of follow-up also likely played a role in the differences among studies, though approximately 86% of studies had more than 2 years of follow-up, suggesting that long-term prediction is possible. Indeed, the results of the subgroup analysis according to the follow-up period generally did not differ from the results of the main analysis. 74% of studies were of high quality, however, subgroup analysis based on study quality did not change the effect size compared to the main analysis, suggesting that study quality was generally sufficient. In addition, even after addressing the considerable heterogeneity using the ‘leave one out’ method, about 25% of the analyses had substantial to considerable heterogeneity while almost 75% did not. Furthermore, the risk of bias was present in only 3/32 analyses. It is essential to keep in mind that the different subtypes of FOG were not considered (e.g., ON versus OFF medication freezing, akinetic vs. trembling type). Also, the limitations of observational studies, as compared to RCTs, should be considered along with the limited number of studies that included potentially intriguing, very strong risk factors (e.g., falls RR:11.8; white matter hyperintensities HR:3.29). Future studies are needed to address these important issues.

Despite these limitations, the current findings based on nearly 9000 subjects with an average follow-up of 4 years suggest that multiple risk factors are linked to the future development of FOG. The present findings and the multivariable analysis suggest that patients who have specific risk factors are more likely to develop FOG and that the risk of FOG incidence is not just a simple reflection of disease severity. While not all of the identified factors are modifiable, many apparently are, at least to some degree (Fig. 4). Therefore, in addition to efforts to ameliorate FOG in those patients who already experience this debilitating symptom, perhaps it is time to shift the paradigm and start to consider a personalized, preventive approach to treating FOG by intervening in patients who have an increased risk, before FOG develops and becomes clinically apparent.

## Methods

### Search strategy, Inclusion criteria and data extraction

A systematic literature search was performed by a qualified librarian in Medline, EMBASE, CINAHL, and Web of Science databases through December 31, 2022, with no year or language limitations. (Additional search strategies are presented in Supplementary Material Methods). Briefly, studies were eligible for inclusion if (a) they used a cohort design (prospective or retrospective), (b) they included adult patients who had received a diagnosis of PD without FOG at baseline, (c) data on possible FOG predictors were measured at baseline, and (d) incident FOG was assessed at follow-up.

Published study-based level data were extracted independently by two investigators (Y.B. and T.H.) and reviewed by a third investigator (SS). Disagreements were resolved via discussion. Reference lists of the included studies were explored for additional studies. No articles needed translation. We included in the meta-analysis studies that were grouped-based level (FOG vs. No-FOG) and their results were presented in the main text or additional information (*n* = 35/38). FOG was generally defined as a “brief, episodic absence or marked reduction of forward progression of the feet despite the intention to walk”^2^. In the included studies, FOG was detected in several subjective ways. These included self-report via (1) a single question “Do you feel that your feet are glued to the floor?”; (2) using the FOG-Q (item 3); or (3) the NFOG-Q questionnaire (“Did you experience Freezing episodes over the past month”), after showing them a video with different kinds of freezing episodes. Objectively, FOG was identified via clinical observation (MDS-UPDRS item 3.11) or a neurological examination by a certified neurologist. The Preferred Reporting Items for Systematic-Reviews and Meta-Analysis (PRISMA) guidelines were followed. The definitions used in the original studies for the identification of FOG and all predictors were accepted. The meta-analysis was pre-registered at PROSPERO, study ID: CRD42022325489.

### Study quality assessment

All included studies were assessed for methodological quality using the Newcastle - Ottawa Scale (NOS) for cohort studies^69^. This tool is based on a system of stars (*) awarded for each criterion that is fulfilled. Quality is assessed on the selection of the sample including the representativeness of the exposed cohort and the selection of non-exposed cohort, ascertainment of exposure (maximum 4 stars), comparability of the cohorts on the basis of study design and analysis (maximum 2 stars) and the assessment of the outcome (maximum of 3 stars). The maximum number of stars is 9; studies were graded as high quality, for scores of 6 and up, and low for scores <6.

### Statistical analysis

Possible predictors were compared between people who developed FOG and those who did not. When available, data on baseline characteristics were analyzed. The standardized mean difference (SMD) was calculated using mean and standard deviation (SD) for continuous variables, and the risk ratio (RR) was calculated using numbers and proportions for categorical variables (see Supplementary Table 1 for a list of abbreviations used). SMDs of 0·2, 0·5, and 0·8 are considered small, medium, and large, respectively^70^. In the absence of baseline information, the Hazard Ratio (HR) was calculated using a log transformation of the effect size, and 95% confidence intervals (CI). Data were meta-analyzed when at least data from two different studies were available. When available, data was stratified by characteristics of the assessment (e.g., on/off medications; drug-naïve at baseline was considered as off medications). A random-effects meta-analysis model was applied to estimate the overall magnitude and statistical significance of an effect. The random-effects model was preferred to a fixed-effects model because the included studies included potential sources of heterogeneity arising from differences in cohorts and analytic methods. The confounding effects of several disease features that are known to be correlated with each other (e.g., age of disease onset, disease duration, motor [MDS-UPDRS part 3] and with non-motor symptoms [depression, anxiety, cognition], and drug therapy) were assessed using a multivariable meta-analyses. Multi-collinearity was assessed using “vif” function in the “metafor” package showing low values (<5) for all variables included in the multivariable analysis. Also, to avoid residual collinearity, the multivariable analysis included only one variable from each domain (e.g., only MOCA or MMSE, but not both in the same model).

Variability between studies was evaluated utilizing the statistical test of homogeneity, I^2^. The magnitude of study heterogeneity was determined according to I^2^ level, where values of <25%, 25–50%, 50–75% and >75% were considered small, moderate, substantial, and considerable^71^. When heterogeneity was deemed considerable, a sensitivity analysis using the ‘leave one out’ (LOU) method was performed, the study suspected as the cause for the high heterogeneity was detected and the random effect size was calculated without it. Publication bias was assessed using visual inspection of Funnel plots (data not shown) and Egger’s regression intercepts, when applicable. Publication bias was considered present when the *p*-value was less than 0.1. Four sub-group analyses were performed: (1) high (or low) quality studies (cut off: score 6), (2) prospective (or retrospective) design, (3) relatively long (or short) follow-up periods (cut off: 2 years), and (4) short (or longer) duration of PD at baseline (cut off: 2 years). Data analyses were performed utilizing R software version 4.1.1 using the ‘meta’, ‘metafor’ and ‘dmetar’ packages. There was no funding source for this study.

### Supplementary information

Suppmlemntary Material

## Data Availability

The relevant meta-analysis data will be available upon reasonable request to the authors.
