# Supplementary material for: A meta-analysis identifies factors predicting the future development of freezing of gait in Parkinson’s disease
Source: NPJ Parkinsons Dis. 2023 Dec 4;9:158. doi: 10.1038/s41531-023-00600-2 (PMC10696025; doi:10.1038/s41531-023-00600-2)

SUPPLEMENTARY MATERIAL METHODS

Search Strategy:

The following search terms were used: 'parkinson* [where * indicates variable text] disease', freez* and gait AND or 'motor block*' or 'start hesitation' or festinat* AND predict* or follow* or prospective* or longitudinal or incid* or onset or shift* or convert*.

Database: Ovid MEDLINE(R) and Epub Ahead of Print, In-Process, In-Data-Review & Other Non-Indexed Citations and Daily <1946 to December 31, 2022>
1 'parkinson* disease'.mp. [mp=title, abstract, original title, name of substance word, subject heading word, floating sub-heading word, keyword heading word, organism supplementary concept waord, protocol supplementary concept word, rare disease supplementary concept word, unique identifier, synonyms]
2 ((freez* and gait) or 'motor block*' or 'start hesitation' or festinat*).mp 3 1 and 2
4 (predict* or follow* or prospective* or longitudinal or incid* or onset or shift* or convert*).mp. or new.ti,ab. 5 3 and 4
****

Embase

#1	'parkinson* disease'
#2	freez* AND gait OR 'motor block*' OR 'start hesitation' OR festinat*
#3	#1 AND #2
#4	predict* OR follow* OR prospective* OR longitudinal OR new:ti,ab OR incid* OR onset OR shift* OR convert*
#5	#3 AND #4

****

CINHAL

S1	parkinson* W1 disease
S2	(freez* AND gait) OR (motor W1 block*) OR (start W1 hesitation) OR festinat*
S3	S1 AND S2
S4	predict* OR follow* OR prospective* OR longitudinal OR incid* OR onset OR shift* OR convert*
S5	S3 AND S4

****

Web of Science

#1	TS=(parkinson* NEAR/0 disease)
#2	TS=((freez* AND gait) OR (motor NEAR/0 block*) OR "start hesitation" OR festinat*)
#3	#1 AND #2
#4	TS=(predict* OR follow* OR prospective* OR longitudinal OR incid* OR onset OR new OR shift* OR convert*)
#5	#3 AND #4 and Articles or Review Articles (Document Types)

Supplementary Table 1: Abbreviation list

Catechol-o-methyl-transferase	COMT
Cerebral spinal fluid	CSF
Confident intervals	CI
Dopamine transporter	DAT
Epworth Sleepiness Scale	ESS
Freezing of gait	FOG
Geriatric depression scale	GDS
Hamilton anxiety rating scale	HAMA
Hamilton depression rating scale	HAMD
Hazard ratio	HR
Hoehn and Yahr stage	H&Y
Levodopa equivalent daily dose	LEDD
Leave one out	LOU
Mini-mental state examination	MMSE
Montreal cognitive assessment	MOCA
Movement Disorder Society, Unified Parkinson's Disease Rating Scale	MDS-UPDRS
New freezing of gait questionnaire	N-FOGQ
Non-Motor Symptom Scale	NMSS
Parkinson’s disease	PD
Parkinson's Disease Questionnaire	PDQ-39
Postural Instability Gait Difficulty	PIGD
Randomized Controlled Trial	RCT
Rapid eye movement	REM
REM sleep behavior disorder screening questionnaire	RBDSQ
Risk ratio	RR
Scales for outcomes in Parkinson's disease	SCOPA-AUT
Standard deviation	SD
Standardized mean difference	SMD
State and trait anxiety inventory	STAI
Trail Making Test	TMT
University of Pennsylvania Smell Identification Test	UPSIT

Supplementary Table 2: Included Studies Quality assessment (NOS)

	Selection				Comparability		Outcome			Overall
	1	2	3	4	1a	1b	1	2	3	
Banks, et al. (2019)	*	*	*	*				*	*	**6**
Chong, et al. (2015)	*	*	*	*				*	*	**6**
Chung, et al. (2020)	*	*	*	*		*	*	*	*	**8**
Chung, Lee, et al. (2019)	*	*	*	*	*	*		*		**7**
Chung, Yoo, et al. (2019)	*	*	*	*		*	*	*	*	**8**
Chung,Yoo, Lee, et al. (2021)	*	*	*	*				*	*	**6**
Chung,Yoo, Shin, et al. (2021)	*	*	*	*	*	*	*	*		**8**
D’Cruz, et al. (2020)	*	*	*	*				*		**5**
D’Cruz, et al. (2021)	*	*	*	*		*	*	*		**7**
Dadar, et al. (2021)	*	*	*	*	*	*		*	*	**8**
Djaldetti, et al. (2018)	*	*	*	*	*	*		*	*	**8**
Ehgoetz Martens, et al. (2017)	*	*	*	*	*			*	*	**7**
Forsaa, et al. (2015)	*	*	*	*				*		**5**
Gallea, et al. (2021)	*	*	*	*				*		**5**
Garcia-Ruiz, et al. (2012)	*	*		*				*		**4**
Giladi, et al. (2001)	*	*	*	*				*		**5**
Herman, et al. (2019)	*	*	*	*			*	*		**6**
Jeong, et al. (2021)	*	*	*	*	*	*	*	*		**8**
Jeong, Lee et al. (2022)	*	*	*	*	*			*	*	**7**
Kelly, et al. (2015)	*	*	*	*	*	*		*	*	**8**
Kim, et al. (2021)	*	*	*	*				*		**5**
Kim, et al. (2018)	*	*	*	*	*	*		*	*	**8**
Kim, et al. (2020)	*	*	*	*			*	*	*	**7**
Kim, Jeon, et al. (2019)	*	*	*	*		*	*	*	*	**8**
Kim, Lee, et al. (2019)	*	*		*				*	*	**5**
Lee, et al. (2021)	*	*	*	*	*	*		*	*	**8**
Li, et al. (2022)	*	*		*				*	*	**5**
Li, et al. (2021)	*	*	*	*	*	*	*	*		**8**
Lo, et al. (2019)	*	*	*					*		**4**
Ou, et al. (2018)	*	*	*	*	*		*	*	*	**8**
Prange, et al. (2019)	*	*	*	*				*	*	**6**
Sarasso, et al. (2022)	*	*	*	*	*	*		*	*	**8**
Tang, et al. (2021)	*	*	*	*		*	*	*	*	**8**
Wang, et al. (2022)	*	*	*	*	*			*		**6**
Wieler, et al. (2016)	*	*	*	*				*	*	**6**
Xu, et al. (2021)	*	*	*	*				*	*	**6**
Zhang, et al. (2016)	*	*	*	*			*		*	**6**
Zhao, et al. (2022)	*	*	*	*			*	*		**6**

Supplementary Table 3: Main meta-analysis results by categories - analyses including only 2 studies

Putative risk factor		N	N		Statistic	Random effects	Heterogeneity	Publication bias
		Studies	FOG	No-FOG		Effect size (95%CI)	LOU effect size (95%CI), I2	
**Demographics and general characteristics**	**Age at baseline**	2	66	237	HR	1.02 (0.98, 1.05)	Substantial	NA
	**PD duration at baseline**	2	75	644	HR	0.99 (0.98, 1.00)	Small	NA
**Motor features**	**Falls**	2	94	176	RR	11.85 (0.83, 168.27)	Substantial	NA
	**MDS-UPDRS Part 3**	2	35	401	HR	1.02 (1.01, 1.03) [a]	Small	NA
	**Timed up and go**	2	33	43	SMD	0.71 (-0.33, 1.75)	Substantial	NA
	**UPDRS Part 3 (ON)**	2	32	34	SMD	0.53 (0.01, 1.04) [a]	Small	NA
	**UPDRS Part 3 (Unknown)**	2	383	832	SMD	0.54 (0.42, 0.67) [a]	Small	NA
**Non motor features**	**Epworth Sleepiness Scale**	2	186	307	SMD	0.47 (0.06, 0.87) [a]	Substantial	NA
	**Hyposmia**	2	286	789	RR	1.26 (0.98, 1.62)	Considerable<br>No LOU analysis (2 studies)	NA
	**NMSS (Chinese version)**	2	340	852	SMD	0.34 (0.09, 0.59) [a]	Substantial	NA
	**UPSIT**	2	202	349	SMD	-0.26 (-0.43, -0.09) [a]	Small	NA
**Cognitive scales**	**Digit backward**	2	61	61	SMD	-0.32 (-0.68, 0.04)	Small	NA
	**Phonetic fluency**	2	61	61	SMD	-0.28 (-0.68, 0.08)	Small	NA
	**Semantic fluency**	2	61	61	SMD	0.26 (-0.26, 0.70)	Moderate	NA
	**Trail Making Test A (sec)**	2	67	119	SMD	0.21 (-0.09, 0.51)	Small	NA
	**Trail Making Test B (sec)**	2	67	119	SMD	0.22 (-0.08, 0.52)	Small	NA
	**Trail Making Test B-A (sec)**	2	67	119	SMD	0.20 (-0.10, 0.51)	Small	NA
**Medication**	**Anticholinergics use (Yes/No)**	2	340	852	RR	1.19 (0.91, 1.56)	Small	NA
	**COMT inhibitors use (Yes/No)**	2	340	852	RR	2.58 (1.51, 4.38) [a]	Small	NA
	**MAOB inhibitors use (Yes/No)**	2	340	852	RR	1.31 (0.96, 1.77)	Small	NA
**Imaging**	**Caudate DAT**	2	61	468	HR	0.84 (0.63, 1.12)	Substantial	NA
**Other**	**CSF Amyloid β42**	2	278	454	SMD	-0.32 (-0.47, -0.17) [a]	Small	NA

Supplementary Table 4: Egger's intercept and p-values

Putative risk factor	N studies	N FOG	N No-FOG	Statistic	Publication bias		
					Eggers' intercept	p-value	Decision
Age at baseline	10	785	1596	SMD	0.325	0.264	No
Age at baseline	2	66	237	HR	NA	NA	NA
Age of PD onset	8	899	1607	SMD	0.2561	0.669	No
Age of PD onset	6	490	2182	HR	0.015	0.475	No
Age of PD onset (early PD=1)	4	450	949	RR	-0.035	0.713	No
Amantadine use (Yes/No)	4	475	984	RR	-0.711	0.786	No
Anticholinergics use (Yes/No)	2	340	852	RR	NA	NA	NA
Berg balance score	3	165	261	SMD	0.03	0.009	Yes
Caudate DAT	4	535	741	SMD	-0.163	0.229	No
Caudate DAT	2	61	468	HR	NA	NA	NA
COMT inhibitors use (Yes/No)	2	340	852	RR	NA	NA	NA
CSF Amyloid β42	2	278	454	SMD	NA	NA	NA
Digit backward	2	61	61	SMD	NA	NA	NA
Dopamine agonist use (Yes/No)	4	475	984	RR	0.238	0.413	No
Education (Years)	7	265	356	SMD	-0.131	0.893	No
Epworth Sleepiness Scale	2	186	307	SMD	NA	NA	NA
Falls	2	94	176	RR	NA	NA	NA
GDS	6	609	799	SMD	0.0137	0.0535	Yes
H&Y	5	199	271	SMD	1.373	0.486	No
H&Y (Level 1)	4	221	413	RR	-0.0663	0.3461	No
H&Y (Level 2)	4	221	413	RR	-0.2025	0.121	No
H&Y (Level 3)	3	153	298	RR	0.722	0.195	No
HAMA	4	356	489	SMD	0.647	0.358	No
HAMD	4	356	489	SMD	0.696	0.268	No
Hyposmia	2	286	789	RR	NA	NA	NA
LEDD	11	763	1505	SMD	0.5336	0.936	No
LEDD	4	199	696	HR	-0.0002	0.855	No
Levodopa use (Yes/No)	4	475	984	RR	0.032	0.143	No
MAOB inhibitors use (Yes/No)	2	340	852	RR	NA	NA	NA
MDS-UPDRS Part 3	2	35	401	HR	NA	NA	NA
MDS-UPDRS Part 3 (OFF)	3	253	368	SMD	0.262	0.772	No
MDS-UPDRS Part 3 (ON)	3	78	130	SMD	0.152	0.1256	No
MDS-UPDRS Part 3 (Unknown)	4	498	807	SMD	0.152	0.125	No
MMSE	4	329	843	SMD	-0.141	0.854	No
MOCA	9	867	1302	SMD	-0.6184	0.1027	No
NMSQuest	3	165	162	SMD	3.128	0.147	No
NMSS (Chinese version)	2	340	852	SMD	NA	NA	NA
PD duration at baseline	14	1117	2037	SMD	0.2722	0.829	No
PD duration at baseline	2	75	644	HR	NA	NA	NA
PD subtype (intermediate)	3	208	279	RR	-1.054	0.718	No

PD subtype (PIGD)	6	504	1048	RR	0.0348	0.4821	No
PD subtype (tremor dominant)	6	504	1048	RR	-0.2403	0.874	No
PDQ39	3	288	755	SMD	0.805	0.37	No
Phonetic fluency	2	61	61	SMD	NA	NA	NA
PIGD score	7	931	1659	SMD	0.6234	0.846	No
Putmant DAT	3	467	626	SMD	0.5809	0.165	No
Putmant DAT	7	312	1441	HR	0.021	0.023	Yes
RBDSQ	4	263	410	SMD	0.1999	0.678	No
SCOPA-AUT	3	228	380	SMD	0.2188	0.4149	No
Semantic fluency	2	61	61	SMD	NA	NA	NA
Sex (Female=1)	21	1571	2949	RR	0.0825	0.132	No
Sex (Female=1)	13	847	4434	HR	-0.134	0.293	No
STAI	5	583	768	SMD	0.108	0.344	No
Timed up and go	2	33	43	SMD	NA	NA	NA
Trail Making Test A (sec)	2	67	119	SMD	NA	NA	NA
Trail Making Test B (sec)	2	67	119	SMD	NA	NA	NA
Trail Making Test B-A (sec)	2	67	119	SMD	NA	NA	NA
Tremor score	6	920	1648	SMD	0.2136	0.335	No
UPDRS Part 3 (OFF)	3	90	112	SMD	0.6478	0.5414	No
UPDRS Part 3 (ON)	2	32	34	SMD	NA	NA	NA
UPDRS Part 3 (Unknown)	2	383	832	SMD	NA	NA	NA
UPSIT	2	202	349	SMD	NA	NA	NA

Supplementary Table 5: Subgroup analyses results

Baseline characteristic	Subgroup	Level	N studies	N		Statistic	Random effects	Heterogeneity	
				FOG	No-FOG		Effect size (95%CI)	I^2 (95%CI)	Magnitude
**LEDD**	Design	Prospective	11	763	1505	SMD	0.54 (0.32, 0.77)	77% (59, 87%)	Considerable
		Retrospective	0						
	Quality	Low	2	58	153	SMD	0.54 (0.32, 0.77)	0%	Small
		High	9	705	1352	SMD	0.59 (0.33, 0.85)	80% (64, 89%)	Considerable
	Follow up	Short	3	337	945	SMD	0.46 (0.15, 0.77)	66% (0, 90%)	Substantial
		Long	8	426	560	SMD	0.58 (0.27, 0.89)	72% (43, 87%)	Substantial
	PD duration	New PD	3	155	152	SMD	0.68 (0.06, 1.30)	69% (0, 91%)	Substantial
		Over 2 Years	8	608	1353	SMD	0.50 (0.25, 0.76)	78% (58, 89%)	Considerable
**H&Y**	Design	Prospective	4	133	179	SMD	0.81 (0.18, 1.44)	82% (53, 93%)	Considerable
		Retrospective	1	66	92				
	Quality	Low	2	83	100	SMD	0.50 (0.20, 0.80)	0%	Small
		High	3	116	171	SMD	0.87 (0.07, 1.67)	86% (60,95%)	Considerable
	Follow up	Short	0						
		Long	5	199	271	SMD	0.74 (0.24, 1.24)	85% (66, 93%)	Considerable
	PD duration	New PD	2	86	112	SMD	0.44 (0.15, 0.72)	0%	Small
		Over 2 Year	3	113	159	SMD	1.05 (0.42, 1.69)	66% (0, 90%)	Substantial
**MDS-UPDRS Part 3 (ON)**	Design	Prospective	3	78	130	SMD	0.59 (0.28, 0.9)	0% (0, 90%)	Small
		Retrospective	0						
	Quality	Low	0						
		High	3	78	130	SMD	0.59 (0.28, 0.90)	0% (0, 90%)	Small
	Follow up	Short	1	41	88				
		Long	2	37	42	SMD	0.42 (-0.02, 0.87)	0%	Small
	PD duration	New PD	0						
		Over 2 Years	3	78	130	SMD	0.59 (0.28, 0.9)	0% (0, 90%)	Small

**MDS-UPDRS Part 3 (OFF)**	Design	Prospective	3	253	368	SMD	0.64 (0.22, 1.07)	85% (56, 95%)	Considerable
		Retrospective	0						
	Quality	Low	0						
		High	3	253	368	SMD	0.64 (0.22, 1.07)	85% (56, 95%)	Considerable
	Follow up	Short	0						
		Long	3	253	368	SMD	0.64 (0.22, 1.07)	85% (56, 5%)	Considerable
	PD duration	New PD	1	142	197	SMD			
		Over 2 Year	2	111	171	SMD	0.86 (0.47, 1.25)	85% (56, 95%)	Considerable
**MDS-UPDRS Part 3 (Unknown)**	Design	Prospective	3	309	620	SMD	0.39 (0.25, 0.53)	0% (0, 90%)	Small
		Retrospective	1	189	172				
	Quality	Low	3	366	574	SMD	0.40 (0.26, 0.53)	0% (0, 90%)	Small
		High	1	132	218				
	Follow up	Short	1	41	154				
		Long	3	457	647	SMD	0.37 (0.24, 0.49)	0% (0, 90%)	Small
	PD duration	New PD	2	325	429		0.38 (0.23, 0.53)	0%	Small
		Over 2 Years	2	173	363		0.39 (0.20, 0.57)	0%	Small
**MOCA**	Design	Prospective	6	562	988	SMD	-0.17 (-0.34, -0.00)	58% (0, 83%)	Substantial
		Retrospective	3	305	314	SMD	-0.30 (-0.51, -0.10)	40% (0, 82%)	Moderate
	Quality	Low	3	366	574	SMD	-0.27 (-0.52, -0.02)	63% (0, 89%)	Substantial
		High	6	501	728	SMD	-0.17 (-0.33, -0.01)	46% (0, 79%)	Moderate
	Follow up	Short	1	41	145				
		Long	8	826	1157	SMD	-0.24 (-0.37, -0.11)	50% (0, 77%)	Moderate
	PD duration	New PD	5	583	768	SMD	-0.35 (-0.46, -0.24)	0% (0, 70%)	Small
		Over 2 Years	4	284	534	SMD	-0.05 (-0.19, 0.10)	0% (0, 85%)	Small
**MMSE**	Design	Prospective	4	329	843	SMD	-0.15 (-0.28, -0.02)	0% (0, 85%)	Small
		Retrospective	0						
	Quality	Low	0						

		High	4	329	843	SMD	-0.15 (-0.28, -0.02)	0% (0, 85%)	Small
	Follow up	Short	2	296	800	SMD	-0.14 (-0.27, -0.01)	0%	Small
		Long	2	33	43	SMD	-0.29 (-0.75, 0.17)	0%	Small
	PD duration	New PD	1	7	12				
		Over 2 Years	3	322	831	SMD	-0.15 (-0.28, -0.03)	0% (0, 90%)	Small
**HAMA**	Design	Prospective	4	356	489	SMD	0.31 (0.09, 0.53)	58% (0, 86%)	Substantial
		Retrospective	0						
	Quality	Low	0						
		High	4	356	489	SMD	0.31 (0.09, 0.53)	58% (0, 86%)	Substantial
	Follow up	Short	0						
		Long	4	356	489	SMD	0.31 (0.09, 0.53)	58% (0, 86%)	Substantial
	PD duration	New PD	1	128	120				
		Over 2 Years	3	228	369	SMD	0.42 (0.25, 0.58)	51% (0, 86%)	Substantial
**HAMD**	Design	Prospective	4	356	489	SMD	0.48 (0.34, 0.62)	0% (0, 85%)	Small
		Retrospective	0						
	Quality	Low	0						
		High	4	356	489	SMD	0.48 (0.34, 0.62)	0% (0, 85%)	Small
	Follow up	Short	0						
		Long	4	356	489	SMD	0.48 (0.34, 0.62)	0% (0, 85%)	Small
	PD duration	New PD	1	128	120				
		Over 2 Years	3	228	369	SMD	0.50 (0.33, 0.66)	17% (0, 91%)	Small
**Berg balance score**	Design	Prospective	3	165	261	SMD	-0.50 (-0.96, -0.05)	58% (0, 88%)	Substantial
		Retrospective	0						
	Quality	Low	0						
		High	3	165	261	SMD	-0.50 (-0.96, -0.05)	58% (0, 88%)	Substantial
	Follow up	Short	0						
		Long	3	165	261	SMD	-0.50 (-0.96, -0.05)	58% (0, 88%)	Substantial

	PD duration	New PD	1	7	12				
		Over 2 Years	2	158	245	SMD	-0.34 (-0.67, -0.01)	39%	Moderate
**PDQ39**	Design	Prospective	3	288	755	SMD	0.71 (0.57, 0.85)	0% (0, 90%)	Small
		Retrospective	0						
	Quality	Low	0						
		High	3	288	755	SMD	0.71 (0.57, 0.85)	0% (0, 90%)	Small
	Follow up	Short	1	255	712				
		Long	2	33	43	SMD	0.56 (0.09, 1.02)	0%	Small
	PD duration	New PD	1						
		Over 2 Years	2	281	743	SMD	0.73 (0.59, 0.87)	0%	Small
**Levodopa use**	Design	Prospective	4	475	984	RR	1.54 (1.06, 2.25)	91% (81, 96%)	Considerable
		Retrospective	0						
	Quality	Low	0						
		High	4	475	984	RR	1.54 (1.06, 2.25)	91% (81, 96%)	Considerable
	Follow up	Short	1	255	712				
		Long	3	220	272	RR	1.79 (1.19, 2.7)	82% (44, 94%)	Considerable
	PD duration	New PD	2	135	132	RR	2.30 (1.77, 2.97)	0%	Small
		Over 2 Year	2	340	852	RR	1.17 (0.98, 1.40)	71% (0, 93%)	Substantial

Supplementary Figure 1: Summary of all putative risk factors for incident FOG according to the type of data available with effect size and 95% confidence interval. In each panel, factors are organized by alphabetic order; A. Standardized mean difference (SMD); B. Relative risk; C. Hazard ratio.

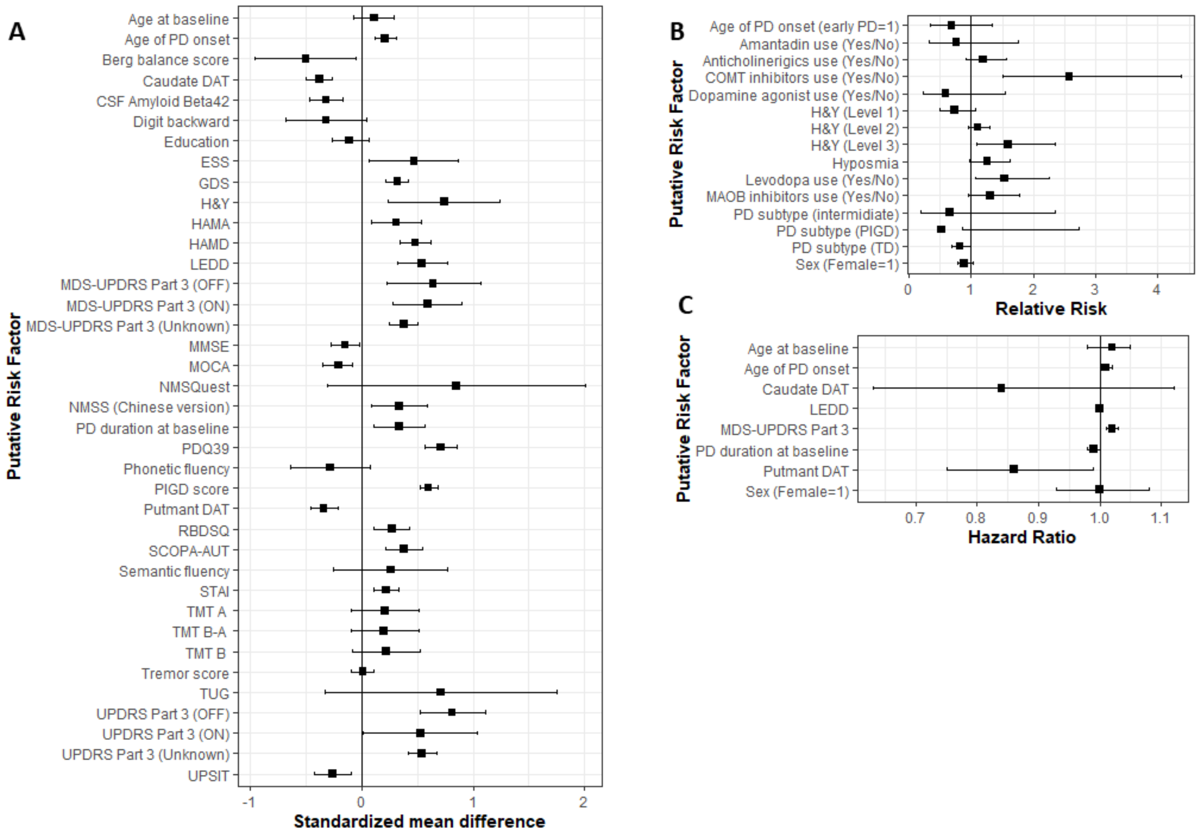

Supplement: Supplementary file 1 — Suppmlemntary Material [file 41531_2023_600_MOESM1_ESM.pdf]
